# The potential impact of coagulation factor XIII in trauma-induced coagulopathy – a retrospective case series analysis

**DOI:** 10.1007/s00068-023-02221-z

**Published:** 2023-01-21

**Authors:** Michaela Wilhelmi, Alexander Albrecht, Christian Macke, Mathias Wilhelmi, Mohammed Omar, Marcel Winkelmann, Jan-Dierk Clausen

**Affiliations:** 1 Trauma Department, Hannover Medical School, Hannover, Germany; 2 Sport Department, Hannover Medical School, Hannover, Germany; 3 Department for Vascular and Endovascular Surgery, St. Bernward Hospital, Hildesheim, Germany

**Keywords:** FXIII, Trauma coagulopathy, RBC transfusion

## Abstract

**Background:**

The role of factor XIII (FXIII) in trauma-induced coagulopathy (TIC) is not fully understood.

**Methods:**

We evaluated FXIII supplementation in severely injured patients with persistent bleeding. This was a retrospective case series analysis.

**Results:**

Twenty-four patients received FXIII concentrate within 24 h of admission for bleeding that continued after transfusion of > 6 U red blood cells (RBCs); control patients (*n* = 27) did not receive FXIII concentrate. Both study groups were similar regarding injury severity score and global coagulation tests, but FXIII activity levels were significantly higher and lactate levels significantly lower in the control group, respectively. The differences in FXIII activity between the groups could be attributed to a more severe trauma-induced coagulopathy in FXIII-deficient patients, as demonstrated by lower fibrinogen and higher lactate levels. The median dose of FXIII concentrate within 24 h of admission was 2500 IU (IQR: 1250–4375). Median 24-h transfusion of RBCs (primary study endpoint) was significantly higher in the FXIII group versus controls (10.0 U, IQR 5–14 U vs. 2, IQR 0–6 U; *p* < 0.01). Subsequently, while patients were in the intensive care unit, there was no statistically significant difference regarding RBC transfusion anymore and the overall clinical outcomes were similar in both patient groups.

**Conclusions:**

The substitution of FXIII in patients who were more seriously compromised due to higher lactate levels and who presented with initially more severe bleedings than patients in the control group, resulted in a comparable transfusion necessity after 24 h. Thus, we guess that the substitution of FXIII in severely injured patients with ongoing bleeding might have an impact on their clinical outcome.

**Supplementary Information:**

The online version contains supplementary material available at 10.1007/s00068-023-02221-z.

## Background

Severe bleeding remains a challenge in the management of major trauma patients and accounts for approximately 50% of deaths [1]. Therefore, early and aggressive treatment of the underlying coagulopathy is mandatory. Surgical measures should be complemented by a balanced transfusion strategy based on the transfusion of red blood cells (RBCs), fresh frozen plasma (FFP) and platelet concentrate (PC). Some European trauma units use viscoelastic testing to guide hemostatic intervention and this may support treatment with coagulation factor concentrates such as fibrinogen concentrate (FC) and prothrombin complex concentrate (PCC) (2; 3).

Coagulation factor XIII (FXIII) – also described as ‘fibrin stabilizing factor’ – is well known to have a physiological role in the processes of fibrin polymerization and clot stabilization, thereby protecting the clot from premature degradation [4–8]. This has led to the hypothesis that FXIII supplementation may be effective for treating excessive bleeding or preventing trauma-induced coagulopathy (TIC). Clinical data analyzing the effects of FXIII supplementation among patients with bleeding complications are sparse. Therefore, we investigated the impact of treatment with FXIII concentrate in a cohort of trauma patients with severe bleeding.

## Methods

**Fig. 1:**
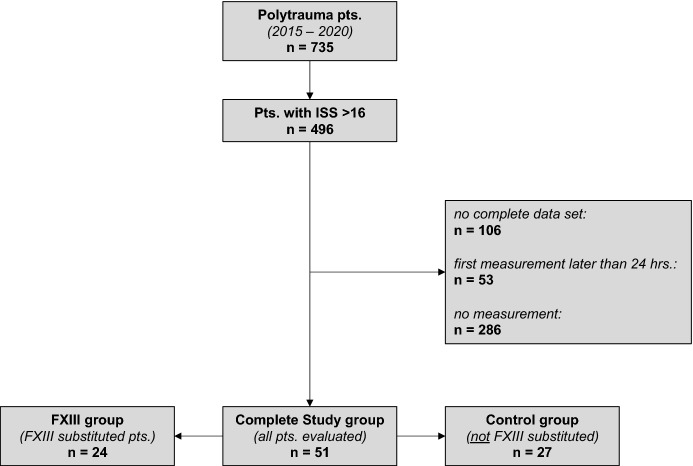
Flowchart describing patient selection due to pre-defined inclusion/ exclusion criteria

In this retrospective study, we evaluated the charts of severely injured trauma patients who were admitted to Hannover Medical School trauma center (certified level one) between 01/2015 and 12/2020. The primary inclusion criteria were age 18–80 years and an injury severity score (ISS) ≥ 16. The main exclusion criteria were the absence of FXIII analyses within the first 24 h, no complete data sets or no FXIII measurements at all (see Fig. 1).

For bleeding management, all patients were treated according to our local standard operating procedure, which is based on commonly known and recommended strategies i.e., a massive transfusion protocol and goal-directed therapy (administration of coagulation factors) on basis of CCA and ROTEM analyses. However, during that time some anesthesiologists began to substitute FXIII in some cases with severely ongoing (> 6 RBCs) and otherwise not manageable bleedings. Since FXIII therapy is not yet evidence-based, this was not a mandatory step. The decision on whether to administer FXIII concentrate was exclusively based on the clinical judgment of the attending anesthesiologist.

Patients who received FXIII concentrate in the early phase of bleeding management (during the first 24 h after admission) were included in the FXIII group. In an attempt to establish a control group, we identified patients of comparable age, sex and ISS.

The primary study endpoint was the amount of RBCs transfused during the first 24 h after admission and later on during the stay in the intensive care unit (ICU). Secondary outcome parameters were the amount of other allogeneic blood products (FFP and PC) and coagulation factor concentrates (PCC and FC) administered. Furthermore, the length of stay in the ICU and deaths during hospitalization were assessed.

Data are presented as the median and interquartile range (IQR), as well as minimum and maximum. Length of stay in the ICU as well as duration of vasopressor therapy are expressed as mean ± SD. The distribution of data was evaluated using the Kolmogorov–Smirnov test. Between-group comparison of normally distributed data was based on the Student’s *t*-test, while a non-parametric test for independent samples with Yates correction was used for data not following normal distribution. All analyses were performed using SPSS Statistics (Version 26, IBM Inc., Armonk, New York). Significant differences were defined as *p* < 0.05.

For evaluation of FXIII activity levels probes of citrated blood were measured with the Berichrom® FXIII assay (Siemens AG, Berlin/Munich/Germany; normal range: 70–140%). Time point 1 (TP1): was between admission and the following 24 h; Timepoint 2 (TP2): thereafter (> 24 h after admission). Prior to all analyses, the study was approved by the local ethics committee at Hannover Medical School, Hannover, Germany.

## Results

As shown in Fig. 1 as well as Table 1, a total of 51 patients were evaluated in this study. Of these, 24 were treated with FXIII concentrate (FXIII group) and 27 did not receive FXIII (control group). The two groups were comparable regarding age, sex and ISS. There were no significant differences in global tests of coagulation (Quick, international normalized ratio (INR) and partial thromboplastin time (PTT) or platelet count upon admission. However, median FXIII activity levels at the time of admission (TP 1) were significantly higher in the control group than in the FXIII group (94 IQR 67.4–101.8% vs. 66.5 IQR 47–78%; *p* < 0.001) and fibrinogen levels were significantly lower in the FXIII group (1.2 IQR 0.91–2 g/L vs. 2.24 IQR 1.79–2.5 g/L; *p* < 0.001). Furthermore, lactate levels were significantly higher in the FXIII group (4 IQR 2.8–4.7 mmol/L vs. 1.6 IQR 1–2.7 mmol/L; *p* < 0.001). The median dose of FXIII administered during the first 24 h in the FXIII group was 2500 IQR 1250–4375 IU.

**Table 1:** Epidemiologic data and baseline values on admission

	Age years	ISS	FXIII %	Fib g/L	Lactate mmol/L	Quick %	PTT sec	Platelet k/ul
Control group								
Median	53	36	94	2.24	1.6	74	37	119
75% Percentile	63	48	101.8	2.5	2.7	77.6	39	150
25% Percentile	37	29	67.4	1.79	1	64	33	103
FXIII group								
Median	45	34	66.5	1.2	4	73	37	111
75% Percentile	63	43	78	2	4.7	81	41	137
25% Percentile	29	34	47	0.91	2.8	65.1	33	88
*p* value	> 0.05	> 0.05	** < 0.001**	** < 0.001**	** < 0.001**	> 0.05	> 0.05	> 0.05

Bold numbers represent all those comparisons with significant differences

*Fib* fibrinogen; *FXIII* factor XIII; *ISS* injury severity score; *PTT* partial thromboplastin time; *platelet* platelet count

Compared with the control group, patients in the FXIII group received significantly more RBCs (10 IQR 5–14 U vs. 2 IQR 0–6 U; *p* < 0.01) and FC (1 IQR 0—4 g vs. 0 IQR 0–2 g; *p* < 0.05) during the first 24 h after admission (Table 2). There were also trends towards increased transfusion of FFP (4 IQR 2 – 9 U vs. 0 IQR 0–5 U; *p* > 0.05) and PCC (1000 IQR 0–2500 IU vs. 0 IQR 0–2000 IU; *p* > 0.05) in the FXIII group versus the control group, but these differences were not statistically significant. After administration of FXIII concentrate, the median plasma activity of FXIII was comparable in both groups (FXIII group: 83.7 IQR 69.8–96.0% vs. control group: 78.1 IQR 59.28–86.63%; *p* > 0.05). Furthermore, blood loss was reduced/bleeding stopped and cardio-circulatory system was stabilized in patients treated with FXIII concentrate.

**Table 2:** Hemostatic therapy administered during the first 24 h after admission

	FFP U	PC U	FC	PCC I.U	RBC U	FXIII concentrate I.U
Control group						
Median	0	0	0	0	2	–
75% Percentile	5	2	2000	2000	6	–
25% Percentile	0	0	0	0	0	–
FXIII group						
Median	4	1	1000	1000	10	2500
75% Percentile	9	4	4000	2500	14	4375
25% Percentile	2	0	0	0	5	1250
*p* value	> 0.05	> 0.05	** < 0.05**	> 0.05	** < 0.01**	n.d

Bold numbers represent all those comparisons with significant differences

*FC* fibrinogen concentrate; *FFP* fresh frozen plasma; *FXIII* factor XIII; *IU* international units; *PC* platelet concentrate; *PCC* prothrombin complex concentrate; *RBC* red blood cell; *U* unit

The mean duration of stay in the ICU was similar in the two study groups (FXIII group: 22.9 ± 11.9 days; control group: 22.6 ± 14.2 days; *p* > 0.05). While in the ICU, patients in the FXIII group received additional treatment with FXIII concentrate (median dose 2500 IQR 1250–3625 IU). Compared with the control group, patients in the FXIII group received significantly more PC and FC while in the ICU (Table 3). However, no statistically significant differences were observed regarding transfusions of RBC and FFP or treatment with PCC. Vasopressor therapy (norepinephrine) was administered for significantly longer in the FXIII group (11.5 ± 9.8 days vs. 7.6 ± 4.6 days; *p* < 0.05). Three patients from each group died during their time in the hospital. No patient of either group developed complications (e.g., thromboembolic events) due to the administration of procoagulant substances.

**Table 3:** Hemostatic therapy administered while patients were in the ICU (after 24 h-end)

	FXIII	FFP U	PC U	FC g	PCC I.U	RBC U	FXIII concentrate I.U
Control group							
Median	78.1	0	0	0	0	6	–
75% Percentile	86.63	1	1	0	1000	10	–
25% Percentile	59.28	0	0	0	0	3	–
FXIII group							n.d
Median	83.7	0	0	0	0	8	2500
75% Percentile	96.0	3	6	0	0	14	3625
25% Percentile	69.8	0	0	0	0	6	1250
*p* value	> 0.05	> 0.05	** < 0.05**	** < 0.05**	> 0.05	> 0.05	n.d

Bold numbers represent all those comparisons with significant differences

*FC* fibrinogen concentrate; *FFP* fresh frozen plasma; *FXIII* factor XIII; *IU* international units; *PC* platelet concentrate; *PCC* prothrombin complex concentrate; *RBC* red blood cell; *U* unit

## Discussion

It has long been known that FXIII acts as the’final’ enzyme of the coagulation cascade. By cross-linking fibrin chains, it stabilizes newly formed blood clots, thereby helping to prevent bleeding complications and to ensure appropriate conditions for wound healing [7]. In recent years, it has become evident that the actions of FXIII are complex and involve a wide variety of additional substrates. In addition to fibrin as the primary target of the action, the enzyme targets antifibrinolytic proteins as well as plasma- and extracellular matrix components. Binding of alpha2-antiplasmin, thrombin-activatable fibrinolysis inhibitor (TAFI) and alpha2-macroglobulin minimizes plasmin-mediated degradation of the fibrin clot, and interactions with molecules such as fibronectin and vitronectin influence and modulate the behavior of blood cells directly involved in tissue repair and remodeling [9]. Consequently, a variety of different disorders are caused by congenital FXIII deficiency, including spontaneous or prolonged post-traumatic bleeding and delayed or deficient wound healing [10–12]. All these aspects have been discussed in detail by Dickneite et al. [6].

Despite the general function of FXIII being well known, detailed understanding of its pathophysiological impact and possible role in treating TIC is lacking. Some clinical studies have suggested that increased FXIII activity levels might be beneficial in the management of surgical patients. For example, in a cardiovascular surgery study, preoperative FXIII supplementation significantly reduced perioperative blood loss and the need for RBC transfusion [13, 14]. In neurosurgical patients, decreased FXIII levels have been associated with an increased risk of postoperative hemorrhage [15, 16]. The impact of FXIII activity in more complex, acute bleeding situations (as frequently observed in major trauma patients), is uncertain as no randomized controlled trials have specifically analyzed FXIII levels or the effects of FXIII replacement therapy. However, some trauma centers evaluated the effect of FXIII substitution in major bleeding situations/ after transfusion of a certain amount of RBCs [2, 3, 17] One major finding, observed in all these studies was a low FXIII activity. Thus, the aim of our current study was to further evaluate the effect and potential impact of coagulation FXIII substitution in patients with severe bleedings.

As in many other trauma centers, the Hannover Medical School trauma center SOP for the management of severe acute bleeding mainly based on commonly known and recommended strategies i.e., a massive transfusion protocol and goal-directed therapy (administration of coagulation factors) on basis of CCA and ROTEM analyses. However, during that time some anesthesiologists began to substitute FXIII in some cases with severely ongoing (> 6 RBCs) and otherwise not manageable bleedings. Notably, and comparable to other studies, e.g. Katzenstensteiner et al., the decision whether to substitute FXIII exclusively based on clinical judgment and independently from FXIII values [17].

In the current study, a retrospective evaluation of laboratory results revealed that patients who received FXIII had significantly reduced FXIII activity levels before treatment. The results suggest that (a) clinical impression and the decision to administer FXIII were correct and (b) FXIII deficiency is associated with higher transfusion requirements over the first 24 h after admission. Following supplementation of FXIII (i.e., when patients were in the ICU), RBC transfusion was not significantly different between the groups, suggesting that treatment might have a positive effect in stabilizing the patients’ coagulation status. This finding is in line with observations made by Katzensteiner et al. who reported a stongly negative correlation between FXIII levels and the amount of RBCs transfused prior to the first FXIII evaluation [17]. The observed necessity for an increased transfusion of FC, PC as well as additional supplementation of FXIII in some patients during the ICU stay might be related to prolonged bleeding due to more pronounced coagulopathy and prolonged vasopressor therapy in the FXIII group.

The processes leading to massive hemorrhage in severely injured patients are complex and cannot be attributed to the failure of a specific pathway of the coagulation cascade as i.e., a too-low FXIII level alone. However, the observation that FXIII supplementation might help to reduce transfusion of RBCs supports the hypothesis that FXIII might play an important role in these processes. The fact that the administration of PCC and FFP was not significantly different between treatment groups further supports this idea. Additionally, Korte’s working group reported data from a large cohort of 1023 patients undergoing non-cardiac surgery where the risk of RBC transfusion was 4.6-fold higher in patients with FXIII activities < 70% [18]. According to the current version of the European trauma guidelines, “monitoring factor XIII levels and replacement below a certain threshold is suggested” [1]. However, no optimal range of FXIII activity or threshold value for supplementation is specified. Updated guidelines from the European Society of Anaesthesiology suggest the administration of FXIII in patients with severe bleeding and FXIII activity level < 30%, while a multimodal algorithm based on two studies of trauma patients recommended FXIII supplementation when activity levels fall below 60% [2, 3, 19]. Furthermore, a review of an expert group suggested a FXIII activity of 60–70% as threshold value for an acquired FXIII deficiency [20]. Considering all these observations and the results of our study we conclude, that it is still not possible to define a clear threshold or at least an activity range below which FXIII should be supplemented. However, due to the observations made in this study, we speculate, that FXIII supplementation may has a positive effect in patients with ongoing severe bleedings. Thus, the clinical condition in combination with low FXIII activity may be more crucial than a certain value alone. Furthermore, Theusinger et al. hypothesized that early intervention may be beneficial, as they observed that FXIII activity decreased between the accident scene and arrival at the emergency room by approximately 20% and directly correlated with ISS [5].

In our study, ISS values were comparable in both groups. However, lactate levels were significantly elevated and fibrinogen levels were significantly reduced in the FXIII group, respectively. This means that FXIII-supplemented patients initially presented with distinct laboratory signs of acidosis and coagulopathy. In other words, although patients in the FXIII group were initially prone to a much higher risk of death, the outcome of both groups was comparable in the end. This observation is supported by Duque et al. who reported on the association between low FXIII activity and long ICU stay as well as high incidences of major bleedings [21].

Main limitation of our study is its`s retrospective character. In this sense, we have to stress that both patient groups evaluated in this study did not perfectly match. Although comparable with respect to age, gender and ISS, initially significantly higher lactate and lower fibrinogen levels indicate that patients of the FXIII group were prone to a much more severe coagulopathy. Furthermore, the risk of bias relating to patient selection or administration of FXIII cannot be completely excluded. Time points for initial as well as later FXIII analyses were not pre-defined. Furthermore, the decision on whether to administer FXIII was based exclusively on clinical judgment and was completely independent of FXIII activity values.. Together with the lack of a sample size calculation, the small number of patients limited the robustness of the study findings, too.

## Conclusions

In our current study, we could observe, that the substitution of FXIII in patients who were more seriously compromised due to higher lactate levels and who presented with initially more severe bleeding than patients in the control group, resulted in a comparable transfusion necessity. Thus, we guess that early substitution of FXIII in severely injured patients with ongoing bleeding might have an impact on their clinical outcome.

“Despite of the results of our current study, there is a clear need for further i.e., large, randomized controlled trials of FXIII supplementation in trauma”

## Supplementary Information

Below is the link to the electronic supplementary material.

Supplementary file1 (PDF 41 KB)

## Data Availability

The data presented in the current study are available from the corresponding author on reasonable request.

## Abbreviations

CCA: Conventional coagulation assays
FC: Fibrinogen concentrate
FFP: Fresh frozen plasma
FXIII: Factor XIII
ICU: Intensive care unit
INR: International normalized ratio
ISS: Injury severity score
PC: Platelet concentrate
PCC: Prothrombin complex concentrate
pts: Patients
PTT: Partial thromboplastin time
RBC: Red blood cell
ROTEM: Rotational thromboelastometry
SD: Standard deviation
SOP: Standard operating procedure
TAFI: Thrombin-activatable fibrinolysis inhibitor
TIC: Trauma-induced coagulopathy

## References

[1] Spahn DR, Bouillon B, Cerny V, Duranteau J, Filipescu D, Hunt BJ (2019). The European guideline on management of major bleeding and coagulopathy following trauma: fifth edition. Crit Care..

[2] Innerhofer P, Fries D, Mittermayr M, Innerhofer N, von Langen D, Hell T (2017). Reversal of trauma-induced coagulopathy using first-line coagulation factor concentrates or fresh frozen plasma (RETIC): a single-centre, parallel-group, open-label, randomised trial. Lancet Haematol.

[3] Stein P, Kaserer A, Sprengel K, Wanner GA, Seifert B, Theusinger OM (2017). Change of transfusion and treatment paradigm in major trauma patients. Anaesthesia.

[4] Schlimp CJ, Cadamuro J, Solomon C, Redl H, Schöchl H (2013). The effect of fibrinogen concentrate and factor XIII on thromboelastometry in 33% diluted blood with albumin, gelatine, hydroxyethyl starch or saline in vitro. Blood Transfus.

[5] Theusinger OM, Baulig W, Asmis LM, Seifert B, Spahn DR (2010). In vitro factor XIII supplementation increases clot firmness in rotation thromboelastometry (ROTEM). Thromb Haemost.

[6] Dickneite G, Herwald H, Korte W, Allanore Y, Denton CP, Matucci CM (2015). Coagulation factor XIII: a multifunctional transglutaminase with clinical potential in a range of conditions. Thromb Haemost.

[7] Levy JH, Goodnough LT (2015). How I use fibrinogen replacement therapy in acquired bleeding. Blood.

[8] von Rappard S, Hinnen C, Lussmann R, Rechsteiner M, Korte W (2017). Factor XIII deficiency and thrombocytopenia are frequent modulators of postoperative clot firmness in a surgical intensive care unit. Transfus Med Hemother.

[9] Richardson VR, Cordell P, Standeven KF, Carter AM (2013). Substrates of Factor XIII-A: roles in thrombosis and wound healing. Clin Sci (Lond).

[10] Ivaskevicius V, Seitz R, Kohler HP, Schroeder V, Muszbek L, Ariens RA (2007). International registry on factor XIII deficiency: a basis formed mostly on European data. Thromb Haemost.

[11] Peyvandi F, Palla R, Menegatti M, Siboni SM, Halimeh S, Faeser B (2012). Coagulation factor activity and clinical bleeding severity in rare bleeding disorders: results from the European network of rare bleeding disorders. J Thromb Haemost.

[12] Duckert F, Jung E, Shmerling DH (1960). A hitherto undescribed congenital haemorrhagic diathesis probably due to fibrin stabilizing factor deficiency. Thromb Diath Haemorrh.

[13] Shainoff JR, Estafanous FG, Yared JP, DiBello PM, Kottke-Marchant K, Loop FD (1994). Low factor XIIIA levels are associated with increased blood loss after coronary artery bypass grafting. J Thorac Cardiovasc Surg.

[14] Gödje O, Gallmeier U, Schelian M, Grünewald M, Mair H (2006). Coagulation factor XIII reduces postoperative bleeding after coronary surgery with extracorporeal circulation. Thorac Cardiovasc Surg.

[15] Gerlach R, Tölle F, Raabe A, Zimmermann M, Siegemund A, Seifert V (2002). Increased risk for postoperative hemorrhage after intracranial surgery in patients with decreased factor XIII activity: implications of a prospective study. Stroke.

[16] Gerlach R, Raabe A, Zimmermann M, Siegemund A, Seifert V (2000). Factor XIII deficiency and postoperative hemorrhage after neurosurgical procedures. Surg Neurol..

[17] Katzensteiner M, Ponschab M, Schöchl H, Oberladstätter D, Zipperle J, Osuchowski M, Schlimp CJ (2022). Factor XIII measurement and substitution in trauma patients after admission to an intensive care unit. J Clin Med..

[18] Listyo S, Forrest E, Graf L, Korte W (2020). The need for red cell support during non-cardiac surgery is associated to pre-transfusion levels of FXIII and the platelet count. J Clin Med..

[19] Kozek-Langenecker SA, Ahmed AB, Afshari A, Albaladejo P, Aldecoa C, Barauskas G (2017). Management of severe perioperative bleeding: guidelines from the European society of anaesthesiology: first update 2016. Eur J Anaesthesiol.

[20] Kleber C, Sablotzki A, Casu S, Olivieri M, Thoms K-M, Horter J, Schmitt FCF, Birschmann I, Fries D, Maegele M (2022). The impact of acquired coagulation factor XIII deficiency in traumatic bleeding and wound healing. Crit Care.

[21] Duque P, Chasco-Ganuza M, Ortuzar A, Almaraz C, Terradillos E, Pérez-Rus G, Pascual C (2022). Acquired FXIII deficiency is associated with high morbidity. Thromb Haemost.

