# Supplementary material for: The potential impact of coagulation factor XIII in trauma-induced coagulopathy – a retrospective case series analysis
Source: Eur J Trauma Emerg Surg. 2023 Jan 21;49(3):1517–23. doi: 10.1007/s00068-023-02221-z (PMC10229685; doi:10.1007/s00068-023-02221-z)
Supplement: Supplementary file 1 — Supplementary file1 (PDF 41 KB) [file 68_2023_2221_MOESM1_ESM.pdf]

Medizinische Hochschule Hannover
Ethikkommission
Vorsitzender: Prof. Dr. Bernhard Schmidt

Sekretariat:
Marion Lange
Telefon: 0511 532-3443

Liane Höft
Telefon: 0511 532-9812

Zeinab Alsakati:
Telefon: 0511 532-6061

Fax: 0511 532-16 3443
ethikkommission@mh-hannover.de

Carl-Neuberg-Straße 1
30625 Hannover
Telefon: 0511 532-0
www.mh-hannover.de

06.01.2022/MLa

MHH Ethikkommission OE 9515
30623 Hannover

Frau
Dr. Michaela Wilhelmi
Klinik für Unfallchirurgie
OE 6230 – im Hause

The potential impact of coagulation factor XIII in trauma-induced coagulopathy – a retrospective case series analysis

Dear Dr. Wilhemi,

As Chairman, on behalf of the members of the Ethics Committee, I took note of your letter dated 22nd December, 2021 and your statements on the above-mentioned project.

According to the principles of the Ethics Committee of the Hannover Medical School neither ethical approval nor informed consent was necessary, as I) data acquisition was retrospective observational within your clinic, II) data were anonymised, and III) the study relied on measurements and data acquisition applied as part of routine care.

Apart from professional regulations, general aspects of data protection law in research projects have to be observed (e.g. the necessary patient informed consent on data use for research purposes).

Sincerely yours,

Prof. Dr. Bernhard Schmidt
Chairman

Dr. Jessica Kaufeld
Managing Physician
